# Perceptions and utilization of generic medicines in Guatemala: a mixed-methods study with physicians and pharmacy staff

**DOI:** 10.1186/s12913-017-1991-z

**Published:** 2017-01-13

**Authors:** David Flood, Irène Mathieu, Anita Chary, Pablo García, Peter Rohloff

**Affiliations:** Wuqu’ Kawoq | Maya Health Alliance, 2 Calle 5-43 Zona 1, Santiago Sacatepéquez, Guatemala

**Keywords:** Generic medicines, Medicine perceptions, Guatemala, Pharmacist roles, Access to medicines, Essential medicines, Non-communicable diseases

## Abstract

**Background:**

Access to low-cost essential generic medicines is a critical health policy goal in low-and-middle income countries (LMICs). Guatemala is an LMIC where there is both limited availability and affordability of these medications. However, attitudes of physicians and pharmacy staff regarding low-cost generics, especially generics for non-communicable diseases (NCDs), have not been fully explored in Guatemala.

**Methods:**

Semi-structured interviews with 30 pharmacy staff and 12 physicians in several highland towns in Guatemala were conducted. Interview questions related to perceptions of low-cost generic medicines, prescription and dispensing practices of generics in the treatment of two NCDs, diabetes and hypertension, and opinions about the roles of pharmacy staff and physicians in selecting medicines for patients. Pharmacy staff were recruited from a random sample of pharmacies and physicians were recruited from a convenience sample. Interview data were analyzed using a thematic approach for qualitative data as well as basic quantitative statistics.

**Results:**

Pharmacy staff and physicians expressed doubt as to the safety and efficacy of low-cost generic medicines in Guatemala. The low cost of generic medicines was often perceived as proof of their inferior quality. In the case of diabetes and hypertension, the decision to utilize a generic medicine was based on multiple factors including the patient’s financial situation, consumer preference, and, to a large extent, physician recommendations.

**Conclusions:**

Interventions to improve generic medication utilization in Guatemala must address the negative perceptions of physicians and pharmacy staff toward low-cost generics. Strengthening state capacity and transparency in the regulation and monitoring of the drug supply is a key goal of access-to-medicines advocacy in Guatemala.

## Background

Access to essential medicines has been on the public health agenda since the 1970s when the World Health Organization (WHO) published the first list of essential drugs and the Alma Ata Declaration proclaimed access to medicines as a pillar of primary health care [1]. Policies to promote drug access initially tended to focus on medicines for acute conditions and infectious diseases. However, in recent years, the rapidly rising burden of chronic non-communicable diseases (NCDs) in low-and-middle income countries (LMICs) has led to calls for improved access to medications for NCDs as well [2, 3]. Surveys conducted in diverse LMICs have shown that essential medicines for NCDs tend to be unaffordable or unavailable in both public and private sectors [4–7].

Access to low-cost essential generic medicines, and, to an increasing degree, generics for NCDs, is thus a critical health policy goal in LMICs. However, barriers to generic access are complex. Demand-side constraints may include health seekers’ negative perceptions of generics or uncertainties regarding medicine quality [8]. On the supply side, numerous factors influence health providers who prescribe and dispense low-cost essential medicines, including efficacy and safety concerns, lack of trust between physicians and pharmacies, misaligned incentives between patient and provider, and pharmaceutical promotional activity [9].

### The pharmaceutical industry and generic medicines in Guatemala

Industry analysts estimate total pharmaceutical sales in Central America in 2014 at US$ 3.7 billion [10]. Guatemala, the most populous country in Central America, is the region’s biggest pharmaceutical market [10, 11]. Although approximately 70% of the US$ 800 million Guatemalan drug market by value is comprised of imported medicines [10], with Mexico serving as the leading source of imports by value as of 2010 [12], there are also 70 domestic and two multinational companies authorized to manufacture medicines within the country [13]. Official documents report that 64% of all drug purchases are made in the private sector, and the vast majority of private health spending is out-of-pocket [14, 15]. Prior research on access to medicines in Guatemala, primarily focused on acute conditions, has suggested that they tend to be unavailable or unaffordable [16–19]. In general, accurate, up-to-date, and publically available information on the pharmaceutical and pharmacy industries in Guatemala is limited.

WHO defines a generic medicine as “a pharmaceutical product, usually intended to be interchangeable with an innovator product, that is manufactured without a license from the innovator company… [and] marketed under a non-proprietary or approved name rather than a proprietary or brand name [20].” In contrast, “originator” or “innovator” medicines are usually defined as the first drugs to be authorized or patented [21]. Generics can be branded or unbranded; a “branded generic” refers to a generic drug sold under a trade name that is different from the originator name. In addition, a few pharmaceutical chains do exist in Guatemala which market “*similares*” (non-bioequivalent copy drugs). These include branches of the Mexican chain Farmacias Similares, which has transformed the private-sector pharmaceutical market in Mexico [22]. However, in Guatemala, the market share for “similares” has remained low compared to the generics market.

In Guatemala, multiple factors impact access to and perceptions of generics. First, Guatemalan intellectual property law offers relatively expansive protection for originator pharmaceuticals relative to peer countries, which delays the introduction of new medicines into the generic market [23–25]. For example, in the case of the long-acting insulin Lantus® (insulin glargine), a generic version would still be restricted in Guatemala even after the drug lost its patent in the United States [26]. Second, as in many other LMICs [27], most generics sold in Guatemala are branded generics [28]. Branded generics in Guatemala are aggressively marketed to capture consumer loyalty, thereby allowing a premium to be charged as compared to unbranded generics [25].

An important factor impacting the perceptions of low-cost generics in Guatemala is concern regarding generic drug quality. Although legal frameworks mandate drug and safety monitoring [13], domestic generic manufacturers operate under laws that are outdated with respect to best manufacturing practices [13, 29]. In addition, the limited data available suggest that there are quality issues in the Guatemalan drug market. For example, in the international Medicines Quality Database, whose results have been published in aggregate form [30], eight of the 37 medicine samples submitted from Guatemala failed to pass a quality test [31]. A series of dissolution studies carried out by students and faculty at San Carlos University in Guatemala showed that, in multiple cases, generic versions of drugs were not bioequivalent to originator products [29]. A 2012 Ministry of Health report found that 5.2% of 960 pharmaceutical quality tests did not comply with quality standards [13].

Robust and competitive generic pharmaceutical markets also require that consumers trust the public institutions tasked with monitoring and protecting the drug supply. In Guatemala, however, there is deep mistrust of the political system [32], and there have been numerous scandals in recent years involving collusion between government officials, business elites, and the pharmaceutical and pharmacy industries. For example, in 2014 alone, four large pharmacy chains were fined for advertising false discounts on medicines [33], 12 international pharmaceutical companies operating in Guatemala were found guilty of colluding to raise the prices of some 500 products [34], and the Guatemalan Social Security Institute (IGSS) entered into a fraudulent pharmaceutical procurement contract that ultimately led to the arrest of high-profile government figures including the director of IGSS and the president of the Central Bank [35]. Taken together, such high-profile scandals reinforce public skepticism of the integrity and safety of the drug supply in Guatemala [25].

**Fig. 1:**
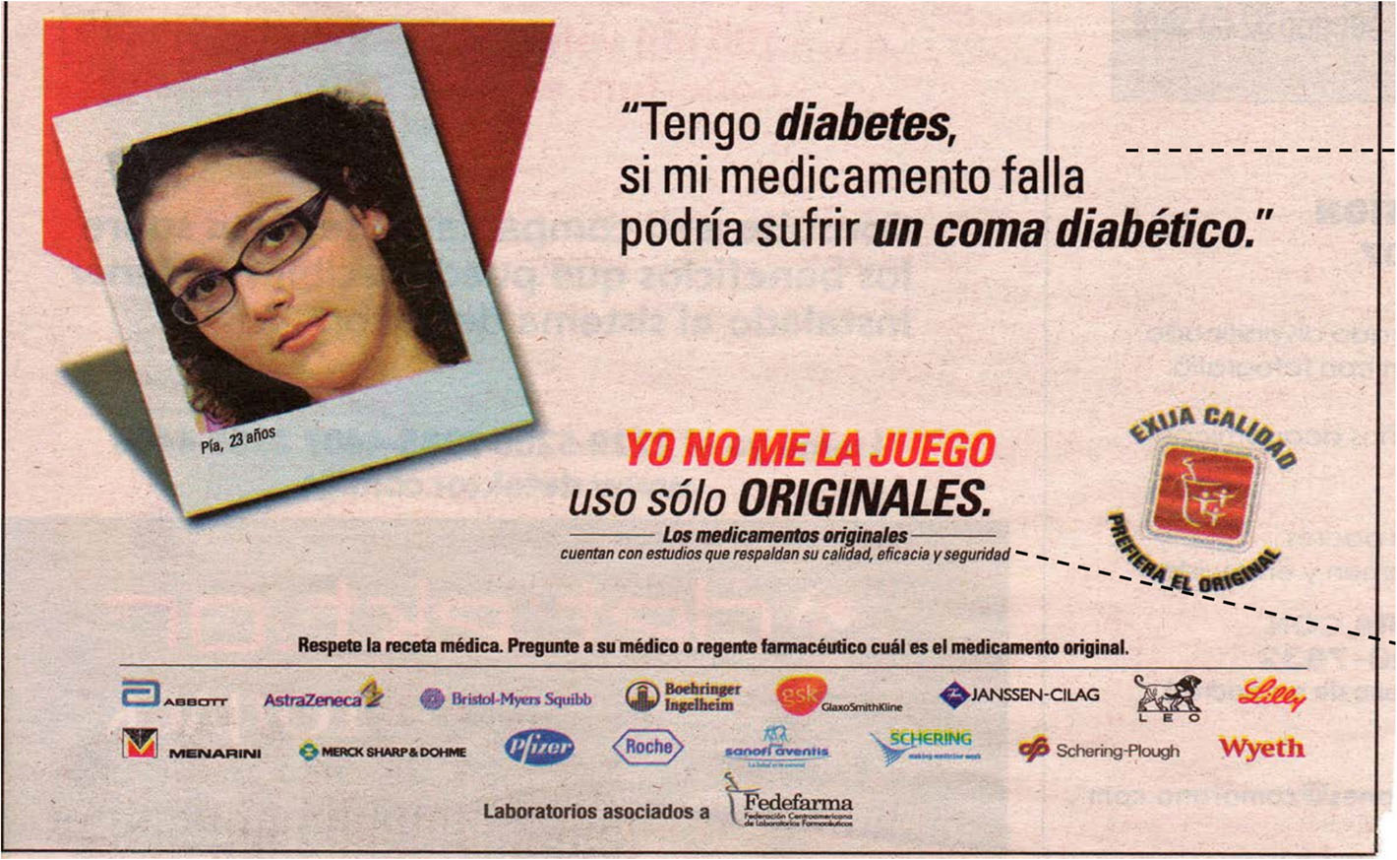
Advertisement in Guatemala promoting originator medicines over generic medicines. In English the text reads: *I have diabetes. If my medicine fails, I could suffer a diabetic coma. I don’t play around; I only use the real thing. Trusted brands [“originator medicines”] have studies that back up their quality, efficacy, and safety. Respect the medical prescription. Ask your doctor or your pharmacist for originator medicines.* Image used with permission of Health Action International

Finally, a multitude of ground-level pressures and incentives tend to favor originator medicines over generics. First, although physicians in Guatemala are legally required to include generic names in prescriptions, such laws are not enforced in practice. In some cases, physicians may steer patients to their own privately-owned pharmacies, creating a financial conflict of interest towards preferentially prescribing and selling higher-cost products like originator medicines [36]. In addition, retail pharmacies commonly advertise discounted or free in-house clinical consultations as a business strategy to increase sales. Finally, while direct-to-consumer advertising in Guatemala is not legally permitted for any prescription medicine [13], the scope of what is considered a prescription drug is very narrow, and, therefore, the vast majority of drugs—including antibiotics—can be purchased over the counter [37]. In practice, consumers are frequently targeted in advertisements for pharmaceutical products [38]. A newspaper advertisement (Fig. 1) published by Fedefarma, a trade group representing more than a dozen well-known international pharmaceutical companies in Central America, illustrates this dynamic well. Fedefarma promotes originator brands to prospective consumers [39] and clinicians [40] by emphasizing themes of quality, safety, and bioequivalence.

Against this background, it remains unclear how the attitudes toward generics of pharmaceutical gatekeepers—prescribing physicians and retail pharmacy staff—impact the access and use of these medicines. One recent study utilized an online survey to demonstrate that many physicians perceived generics to be of lower quality than originator brands, while, at the same time, their prescribing practices were influenced by the affordability of such medicines for patients [41]. However, this study did not examine how other factors such as how interaction with pharmaceutical representatives may also impact perceptions. Additionally, no studies have examined the perceptions of pharmacy staff towards generic medications or how pharmaceutical gatekeepers view generic NCD drugs.

These are important gaps in knowledge since medicines in Guatemala are largely purchased as an out-of-pocket household expense at pharmacies [14, 15]. Even in the public sector, medicines are often out of stock, and, thus, public providers may write prescriptions for patients to purchase medicines privately. Many health seekers eschew clinical providers altogether and directly seek care at pharmacies [37, 42–44]. In this setting, pharmacy staff may theoretically steer patients away from generics or “upsell” originator products.

To examine these issues, we undertook a mixed-methods study (1) to explore physician and retail pharmacy staff’s perceptions regarding generic and medicines and (2) to investigate how these perceptions influence on-the-ground prescribing and dispensing decisions of generic medicines including drugs for NCDs.

## Methods

### Institutional context and ethics

This study was facilitated by Wuqu’ Kawoq | Maya Health Alliance, a non-governmental organization (NGO) working to provide health services, including services for NCDs, in rural indigenous Maya communities in Guatemala. This study was reviewed and approved by the Institutional Review Boards of Wuqu’ Kawoq | Maya Health Alliance and Partners Healthcare, Boston. All participants provided verbal informed consent.

### Overview of methods

The study consisted of semi-structured interviews conducted with 42 physicians and pharmacy staff in three locations in central Guatemala in the province of Chimaltenango: San Juan Comalapa (population 42,200), Tecpán (population 81,100), and the provincial capital Chimaltenango (114,400). These towns were selected as study sites because they represented large concentrations of patients with NCDs for the sponsoring NGO.

### Sample recruitment

Thirty retail pharmacy staff were recruited to the study through random sampling of pharmacies in San Juan Comalapa, Tecpán, and Chimaltenango. “Pharmacy staff” was defined broadly to include any pharmacy worker who dispensed medications to clients, regardless of level of training or professional certification. People in San Juan Comalapa and Tecpán commonly purchase medicines both locally and in the city of Chimaltenango, due to its central location along the major highway leading to both towns. In each of these three towns, the research team created a comprehensive map of all pharmacies and classified each pharmacy in one of five categories: (1) national pharmacy franchise, (2) discount local pharmacy, (3) non-discount local pharmacy, (4) hybrid clinic-pharmacy; or (5) government-subsidized pharmacy (through the national Program for Medication Accessibility [PROAM]). In each town, three pharmacies were randomly selected within each category. In San Juan Comalapa and Tecpán, fewer than three pharmacies were present for some categories; in these cases, all pharmacies within a town’s category were selected. In total, across the three locations, interviewees were recruited from 30 distinct pharmacies whose classifications are reported in Table 1A.

**Table 1:** Characteristics of pharmacies and physicians surveyed

A. Pharmacy characteristics (*n* = 30)	Number (percent)
Town of pharmacy surveyed	
San Juan Comalapa	8 (27%)
Tecpán	8 (27%)
Chimaltenango	14 (47%)
Category of pharmacy (*n* = 30)	
National pharmacy franchise	6 (20%)
Discount local pharmacy	7 (23%)
Non-discount local pharmacy	8 (27%)
Hybrid clinic-pharmacy	5 (17%)
Government-subsidized pharmacy	4 (13%)
B. Physician characteristics (*n* = 12)	
Town of physician clinic surveyed	
San Juan Comalapa	5 (42%)
Tecpán	5 (42%)
Chimaltenango	2 (17%)
Category of practice environment	
Public	3 (25%)
Private	6 (50%)
Private and public	2 (17%)
NGO	1 (8%)

Twelve physicians were recruited to the study from San Juan Comalapa, Tecpán, and Chimaltenango. Convenience sampling was used. The research team identified and contacted physicians in all three locations through referrals from health professionals working in San Juan Comalapa and Tecpán. In Chimaltenango, the research team performed site visits to clinics where the sponsoring NGO’s patients commonly reported accessing care. Across the three locations, physician participants were recruited from 12 distinct practice environments as reported in Table 1B.

### Data collection

Semi-structured interviews lasting 20–45 min were conducted with pharmacy staff and physicians in their place of work. All interviews were conducted in Spanish. Interviews with pharmacy staff focused on training, perceptions of generic medicines, prescription practices regarding medicines for diabetes and hypertension, advice given to clients with diabetes and hypertension, and opinions about the roles of pharmacy staff and physicians in care delivery. Interviews with physicians focused on perceptions of generic medicines including generics for diabetes and hypertension, prescribing practices, and opinions about the roles of pharmacy staff and physicians in care delivery. We opted not to define “originator medicines,” “branded generics,” or “unbranded generics” to research participants given that such definitions can be technically complex and that these terms are not used regularly in clinics and pharmacies in Guatemala. As we were most interested in the perceptions and utilization of low-cost generics, which in Guatemala generally refer to unbranded generics, we used the term “generic” in research questions in contrast to “commercial brands,” as these are the terms most commonly used and understood by participants in this setting. Detailed notes were taken during and after each interview, and these serve as the basis for analysis.

### Data analysis

Qualitative data from interviews was analyzed using an inductive approach, allowing dominant themes to emerge from interview notes. Lists of responses to each open-ended question were compiled in matrices organized by participant type. Responses were then coded into thematic categories by one researcher [IM]. Other team members collectively reviewed and arrived at consensus about the application of codes. Finally, as it was hypothesized that pharmacy staff and physicians’ perspectives might differ across interview topics, coded responses in the matrices were compared across participant type. Quantitative data derived from interviews were first entered into a spreadsheet and then imported into Stata (StataCorp; College Station, TX) for analysis. In addition to descriptive statistics, Fisher’s exact test was used to compare categorical responses between physicians and pharmacy staff.

## Results

### Demographics and education

Thirty pharmacy staff and 12 physicians were interviewed. Demographic characteristics of participants are summarized here and in Table 2. Of the 12 physicians sampled, eight were general practitioners who had completed medical school but no further training, three had completed post-graduate residency programs, and one had received a master’s degree in epidemiology. Ten participants attended medical school in Guatemala and two in Cuba.

**Table 2:** Demographic characteristics of participants

	Pharmacy staff	Physicians
Participants (*n*)	30	12
Formal Education level, *n* (%)	Elementary school: 2 (7) Middle school: 9 (30) High school: 17 (57) Post-high school: 2 (7)	Medical school: 8 (67) Residency: 3 (25) Master’s degree: 1 (8)
Pharmacy Specific Training		
No training, *n* (%)	15 (50%)	N/A
Mean duration of training, months ± SD	5.4 ± 8.5	N/A
Pharmacy certification, *n* (%)	7 (23%)	N/A
Mean Work experience, years ± SD	7.3 ± 5.7	13.5 ± 10.4

Among pharmacy staff, the level of formal education prior to entering the pharmacy business varied widely but was typically low, with only two of 30 respondents having received any formal post-high school education. When pharmacy staff were queried as to why they had entered the retail pharmacy field, they expressed a diverse range of motivations, including professional interest in the field, desire to help people, previous work in health care (such as nursing or social work), or economic necessity. Participants often cited social connections as drawing them into the profession, such as having a neighbor, family member, or spouse who worked in a pharmacy practice.

Level of pharmacy-specific training or certification also varied widely. Half of participants had received no pharmacy-specific training of any kind. Among those who did report receiving such training, the quality and nature of the training was heterogeneous. Some reported training that consisted of “reading pharmacy books” while others had engaged in on-the-job apprenticeships within pharmacies or clinics. Some participants referred to prior experience with medications, gained from working in a different health care environment, such as a nursing home or clinic. Among those who self-reported any kind of pharmacy-specific training, the duration of that training was 5.4 ± 8.5 months. Only seven (23%) self-reported earning a license to practice in a pharmacy setting.

### General perceptions of generic cost, safety, and efficacy

Pharmacy staff and physicians’ perceptions of the safety and efficacy of generic medicines are summarized in Table 3. In general, physicians were more likely than pharmacy staff to perceive generic medications to be less safe or less effective. However, these differences in perceptions were not statistically significant, likely due to our small sample size.

**Table 3:** Perceptions of safety and efficacy by pharmacy staff and physicians

	Pharmacy staff (*n* = 30)	Physicians (*n* = 12)	*P*-value
Generics are not as safe as branded drugs	12 (41%)^a^	6 (55%)^a^	0.50^b^
Generics are not as effective as branded drugs	10 (33%)	6 (55%)^a^	0.29^b^

^a^A few respondents answered “do not know” and their answers are excluded from this calculation

^b^Fisher’s exact test

Among pharmacy staff, 41% believed that generics were not as safe as drugs with commercial brand names, whereas 33% believed that they were not as effective. When asked to elaborate on their perceptions of generic drugs, nine pharmacy staff cited “inferior manufacturing ingredients” or “low quality.” They also cited specific examples of clients who had experienced different therapeutic responses, such as changes in blood pressure or glycemic control, when switching between branded and generic medications as evidence of generics’ inferiority. Eight participants reasoned that generic medicines’ low cost was in itself proof of low quality, explaining that because they are “too economical” or “cheap,” generic medicines could not possibly have equal effectiveness. Five participants were concerned by reports they had read in the popular press or trade journals about the heterogeneous quality of various companies’ manufacturing processes. Nine pharmacy staff reported that the distinction between generics and “branded medications” was the branding process itself; “the only difference is the brand because the quality is the same,” as one respondent remarked. One participant distinguished between generic medicines made by trusted manufacturers and “generic generics,” which the participant deemed to be less reputable.

Pharmacy staff who worked at a national pharmacy franchise or a for-profit hybrid clinic-pharmacy were more likely to view generics as less safe than those who worked at community or public pharmacies (26% vs. 70%, *p* = 0.046). A similar trend effect was observed for the perception of efficacy but was not statistically significant (21% vs. 55%, *p* = 0.11).

Among physicians, 55% believed that low-cost generics were not as safe as “branded drugs,” and 55% also stated that they were not as effective. Nine participants expressed at least some reservations about the use of generic medications, with typical comments including that “they are not adequately manufactured,” “they don’t have good quality control,” and “they don’t offer the same results to the patient.” Physicians also acknowledged the heterogeneity of generic drug quality, which was perceived to be dependent on the reliability of the manufacturer. Like pharmacy staff, some physicians reasoned that the low cost of generic drugs was itself indicative of inadequate quality.

When asked about the differences between generic and non-generic drugs, physicians cited prestige in addition to safety and efficacy concerns. One physician explained that patients feel “proud” to be able to pay for expensive drugs. Three physicians described prescribing low-cost generic medicines and subsequently switching to a more expensive drug with a brand name when the generic version was perceived to be ineffective. One participant explained that patients with previous exposure to high-cost drugs with brand names would not respond as well when “downgraded” to a low-cost generic version.

### Interactions with pharmaceutical company representatives

Pharmacy staff and physicians were asked about their interactions with pharmaceutical representatives. Twenty-six of 30 pharmacy staff reported visits from pharmaceutical representatives. Seven said that they had received incentives from representatives in the form of drug samples, price discounts, pens, notebooks, calendars, prizes, and informal presentations. Nineteen pharmacy staff cited pharmaceutical representatives among the resources they used to learn about the latest pharmaceutical products.

Responses were similar among physician participants. All but two of the 12 physicians interviewed said that pharmaceutical sales representatives regularly visited them. Of these 10, four reported receiving incentives from pharmaceutical representatives, including drug samples, electronic office equipment, payment of registration for conferences, calendars, and prescription pads. When asked how they stayed up-to-date on current medical literature, eight physicians cited information provided by pharmaceutical companies and their representatives among other resources. No statistically significant relationship was observed between interactions with pharmaceutical representatives or the receipt of gifts from representatives with perceptions of safety or efficacy of generics for either pharmacy staff or physicians.

### Generic drug utilization for diabetes and hypertension

In the case of diabetes and hypertension, prescribing practices of physicians and dispensing practices of pharmacy staff varied widely and were influenced in part by perceptions regarding the efficacy and safety of low-cost generics. When pharmacy staff were asked how they decide whether to dispense low-cost generic medicine for a patient with a chronic disease, they were in general agreement that it depended on patients’ financial resources and the severity of the condition, with wealthier and sicker patients less likely to be dispensed generics. Many pharmacy staff also emphasized that the decision to use a generic was ultimately up to the patient. As one participant commented, regarding chronic disease medicines, “I tell them, do you want the expensive one or cheap one? Some people die because they don’t want to buy a generic medicine.” One pharmacy worker added that, in the case of chronic diseases, it was the role of pharmacies “to educate people, so they don’t get tricked by brand-name marketing.”

Three physicians cited the patient’s clinical history and prior drug regimens as important factors in deciding what to prescribe. For instance, one participant explained that a more severe clinical presentation or a “very chronic disease” would call for a non-generic medicine, although starting with a generic version and switching if it were ineffective might be acceptable practice. Several participants expressed that concerns regarding the effectiveness and safety of generic medicines influenced their clinical practice. One physician commenting on chronic disease medicines stated, “To prescribe generics is to play with the life of the patient.”

### Roles of physicians and pharmacy staff in providing chronic disease care

The majority of pharmacy staff (87%) stated that a prescribing physician should determine the medicines for diabetes or hypertension. Similarly, most pharmacy staff reported that it is more common for patients with diabetes or hypertension to arrive at the pharmacy with a physician prescription in hand than to ask for a treatment recommendation at the pharmacy. Only 27% of pharmacy staff reported dispensing a medication other than the prescribed medication to a patient with diabetes or hypertension.

In addition to dispensing medications, most pharmacy staff described providing additional medical counseling to patients with diabetes and hypertension. Eighty percent of participants reported offering advice to patients with diabetes and 63% reported giving advice to patients with hypertension. Pharmacy worker advice for managing diabetes included exercise and decreasing the intake of sugar, sugary foods, and fats. In addition to pharmaceuticals, pharmacy staff participants also recommended vitamins, herbs, and electrolyte solutions specifically marketed for patients with diabetes. When providing advice for hypertension, pharmacy staff similarly emphasized increasing physical activity, making dietary changes, and improving psychological well-being. Participants also suggested natural remedies including chamomile tea and garlic with honey.

When physicians were asked about the overall role of pharmacy staff in patient care, several key themes emerged. In particular, interviewed physicians expressed concern about the lack of formal training among pharmacy staff as well as their competing desire to broker the sale of medicines. Physicians frequently stated that the role of pharmacists should be to dispense the medicines as prescribed by the physician without substitutions and to provide education about medicines.

## Discussion

Through semi-structured interviews with physicians and pharmacy staff in Guatemala, this study explored perceptions of low-cost generic drugs in clinical practice and how these perceptions of pharmaceutical gatekeepers impact prescribing and dispensing decisions. Our results offer several insights regarding use and access to low-cost generic medicines, including generics for NCDs, that merit further discussion.

First, both physicians and pharmacy staff expressed doubt as to the safety and efficacy of low-cost generic medicines in Guatemala, in part due to concerns about low manufacturing standards and quality control, which influenced their daily prescribing and dispensing practices. Our results echo studies from other LMICs where physicians and pharmacy staff have expressed mixed or negative perceptions regarding the safety and efficacy of generics [45–48]. One review examining physician perspectives of generic drugs found that doctors in LMICs tend to have less positive views of generics compared to doctors in high-income countries, who had more positive perceptions [49]. Another review of both physician and pharmacist perceptions of generics similarly distinguished between the high trust of generics in mature health systems and the low trust in generic quality, manufacturing, and bioequivalence in less mature health systems [50].

Second, we found that both physicians and pharmacy staff in Guatemala utilize cost as a heuristic for the quality of the medicines they prescribe and dispense. The use of cost as proxy for the quality of pharmaceutical products has been previously reported in Guatemala and in other LMICs. Indeed, there is some evidence that price *does* at times serve as an indicator for quality; a recent study of essential medicines from private pharmacies in 17 LMICs found that failing drugs were on average priced 13.6–18.7% lower than non-failing drugs [51]. Some commentators writing about Guatemala from the field of anthropology have argued that high-priced health care products serve not only to emphasize perceptions of quality but also to appeal to a desire for social status and upward mobility [52, 53]. In the present study, this viewpoint is buttressed by the response of some participants, such as the physician who remarked that some patients “feel proud” to pay for expensive drugs. Furthermore, others have shown that the commercial aspects of medications, such as pricing, may influence the placebo effect [54], and it is therefore reasonable to speculate that cost may indeed affect clinical outcomes independent of a difference in intrinsic efficacy between products.

Third, our results show that physicians, pharmacy staff, and consumers all play a role in determining whether a low-cost generic is ultimately utilized in practice for NCDs. The incentives of each of these actors and the forces that influence them are complex, yet policy implications emerge. We report that pharmacy staff generally, though not always, defer to physician prescriptions, which points to a need to enforce laws requiring generic name in prescriptions and otherwise improve communication between prescribers and dispensers. We also found that pharmaceutical representatives frequently visit physicians and pharmacy staff in Guatemala and provide education about new pharmaceuticals. The ubiquity of these visits and the use of gifts and prizes call attention to a need for a well-articulated government policy regarding interactions between pharmaceutical representatives and providers. Although our study did not show a statistically significant relationship between perceptions of generics and interactions with pharmaceutical representatives, the small sample size and very low numbers of recruited participants who did not interact with pharmaceutical representatives means that our study was significantly underpowered to detect a difference.

Fourth, our results show that pharmacy staff generally have low levels of education and training, yet they play an important role in educating patients about medicines and chronic diseases like diabetes and hypertension. Although this study was not designed to evaluate pharmacy worker decision-making, there is a significant literature showing that pharmacy staff in many LMICs [55, 56], including Guatemala [57, 58], do not deliver treatment advice in accordance with commonly-accepted medical standards. However, in this study, pharmacy staff reported that physicians should be the gatekeepers who determine diabetes and hypertension medicines for patients, that it was more common for patients with diabetes and hypertension to present with physician prescriptions than to ask for advice at pharmacies, and that they did not frequently switch out physician prescriptions. These finding are at odds with the existing literature in the region, which, focusing largely on medications for acute illnesses, finds that most dispensing occurs without the involvement of a prescribing physician [37, 43, 44, 57, 59].

Although these results are subject to methodological bias, they are surprising and unexpected, and they require confirmation and exploration in a larger study. One possibility is that the culture of pharmaceutical dispensing is slowly changing in Guatemala, with a move towards more formal reliance on physician prescriptions. Along these lines, it is worth noting that some key studies of pharmaceutical dispensing in the region are now more than 10 years old [57, 58], and so it is likely time to revisit the issue, including reanalyzing dispensing practices for antibiotics and other acute illness medications. Alternatively, it may be that informally-trained pharmacy workers are simply less comfortably dispensing medications for chronic diseases. This could be because of relative limitations in their knowledge about these medications, exacerbated by the rapid diversification of and growth in products in the chronic illnesses market, which are rapidly outpacing the relatively more stable market for antimicrobials, analgesics, and the like. We also hypothesize that the higher cost of medications for chronic illness is a contributory factor. Given the higher financial stakes, it may be that clients or dispensing practitioners increasingly prefer to formalize transactions with the aid of a written physician prescription.

From a broader perspective, previous literature outlines four main categories of barriers to the implementation of pro-generic policies in LMICs: (1) legal barriers; (2) management and institutional barriers; (3) behavior, perception, and knowledge barriers; and (4) financial barriers [9]. This study principally addresses the third category—in particular, the perceptions of physicians and pharmacy staff and how these perceptions influence clinical behavior. Our results indicate that improving attitudes toward low-cost generics is an important goal. At the same time, as we have detailed in the introduction to this article, there is compelling evidence in Guatemala to support the belief that the safety and efficacy of the drug supply cannot be guaranteed. Indeed, the issue of substandard and counterfeit medicines is a concern in many LMICs [30, 60–63]. In the Guatemalan setting, where regulatory information is limited and drug quality is uncertain, it is thus not surprising that the high price of trusted pharmaceutical brands is therefore used as a surrogate marker for quality.

Taken in context, our results also underline the fact that interventions to improve the perception of low-cost generics and increase their utilization by physicians and pharmacy staff would be insufficient without commensurate efforts to strengthen the regulation and transparency of the drug supply. Unfortunately, in Guatemala’s current political climate, it is difficult to imagine any increased state capacity for regulating or ensuring generic drug supplies in the near future. However, we do see two potential promising avenues. First, in recent years, the Guatemalan judicial system, reinforced by backing from the United Nations’ International Commission against Impunity in Guatemala (CICIG), has made considerable inroads in fighting high-level government corruption, including in the health care sector [64]. We hope that CICIG’s activities will reduce high-profile drug procurement scandals, such as those we mentioned in this paper’s Introduction, and may help to increase public confidence in generic medications. Second, we can see a role for voluntary self-organization and self-regulation by generics manufacturers. Commitments to increased transparency and accountability, for example through reporting on compliance with international manufacturing standards and bioequivalence testing, could go a long way both in improving the quality of the generics drug supply and confronting the allegations of inferiority in both the popular press and the promotional materials of originator brands. Such commitments would no doubt come at increased production costs, but these could potentially be offset as public opinion improved and sales increased.

### Study limitations and future directions

This study has several methodological limitations that limit the generalizability of our findings. First, the sample size of physicians and pharmacy staff was small with limited power to detect some important differences, especially regarding interaction with pharmaceutical representatives. Physicians were recruited based on a convenience sampling strategy that could have introduced bias. Second, geographically, participants were concentrated in one particular region of Guatemala, and their opinions may not reflect the views of physicians and pharmacy staff throughout Guatemala. In particularly, the study did not sample participants in Guatemala City, which is the country’s dominant urban area from both a population and economic perspective, nor did we consider the self-reported ethnicity of providers or pharmacy staff themselves in creating our sample. Additionally, as we did not define “originator medicines,” “branded generics,” and “unbranded generics” to research participants, we are unable to make firm distinctions among the perceptions of drugs in each of these categories. The “branded generic conundrum” has emerged as a primary challenge of the generic medicine policy agenda [63]. Further inquiry into the perception, price, and utilization of branded generics in Guatemala in relation to originator products and unbranded generics is an important future research goal for our team. We also intend to explore in greater detail the unexpected findings about reluctance to dispense medications for chronic illness without a prescription, and to investigate more closely the relationship between prescribing practices and interactions with pharmaceutical representatives. In this regard, the frequency of interaction with pharmaceutical representatives we measure in this study will permit sample size calculations for an adequately powered subsequent study.

## Conclusion

This study demonstrates that physician and pharmacy staff’s perceptions of low-cost generics influence the utilization of these medicines in clinical practice, especially in relation to the treatment of NCDs like diabetes and hypertension. Interventions to improve the perception of generics and increase their utilization by physicians and pharmacy staff are critical. At the same time, additional research on changing dispensing practices of medications for both acute and chronic illnesses is needed, as is further investigation of the impact of interactions with pharmaceutical representatives on prescribing and dispensing practices. Although short-term prospects for increasing state capacity for regulation and monitoring of the drug supply are limited, we anticipate that the emergent anti-impunity movement in Guatemala will increase public trust in generic medications by reducing high-profile scandals over drug procurement schemes. Furthermore, we suggest that the generics industry, through self-regulation and self-organizing, could improve the quality of the generics market while simultaneously increasing their market share.

## Abbreviations

CICIG: Comisión Internacional contra la Impunidad en Guatemala, International Commission against Impunity in Guatemala
IGSS: Instituto Guatemalteco de Seguro Social, Guatemalan Social Security Institute
LMIC: Low- and middle-income country
NCD: non-communicable disease
NGO: non-governmental organization
PROAM: Programa de Acesibilidad a Medicamentos, Program for Medication Accessibility
WHO: World Health Organization
